# Chronic Lymphocytic Leukemia with Translocation (2;14)(p16;q32): A Case Report and Review of the Literature

**DOI:** 10.1155/2016/9037436

**Published:** 2016-11-03

**Authors:** Francisco Socola, Giovanni Insuasti-Beltran, Rodolfo Henrich Lobo, Shebli Atrash, Appalanaidu Sasapu

**Affiliations:** ^1^Department of Medicine, Division of Hematology Oncology, University of Arkansas for Medical Science, Little Rock, AR, USA; ^2^Department of Hematopathology/Molecular Genetics Pathology, University of Arkansas for Medical Sciences, Little Rock, AR, USA

## Abstract

We report the case of a young African American male with no significant past medical history presenting with low back and bilateral leg pain; presenting CBC and chemistries revealed elevated white blood cell count of 250,000, with anemia (Hb 6.8 g/dL) and thrombocytopenia (platelets 9 K/*μ*L), and elevated LDH, 1008. Physical examination findings were notable for diffuse lymphadenopathy and lower extremity skin nodules. Interestingly the bone marrow biopsy revealed involvement by CLL/SLL with translocation (2;14)(p16;q32) and trisomy 12. The patient was treated with fludarabine-based chemotherapy and steroids for CLL-related ITP with excellent response. After three cycles of chemotherapy, all the enlarged lymph nodes and skin nodules disappeared, and patient had achieved complete hematologic response. In this paper we also reviewed the available literature of CLL patients with translocation (2;14).

## 1. Introduction

Chronic lymphocytic leukemia/small lymphocytic lymphoma (CLL/SLL) is the most common type of leukemia in adults, and it is characterized for having chromosomal abnormalities in up to 80% of patients. Among them, deletions of 11q, 13q, 17p, and trisomy 12 have a known prognostic value and play an important role in CLL pathogenesis and evolution, determining patient's outcomes and therapeutic strategies. Many B-cell lymphomas are characterized by chromosomal translocations that involve the immunoglobulin genes; however, chromosome translocations including* IGH* rearrangement on 14q32 were relatively infrequent in CLL/SLL, with a frequency of 4% [1].

The B-cell CLL/lymphoma 11A* (BCL11A)* gene is located in chromosome 2p16.1 and encodes a zinc finger protein that interacts directly with* BCL6*, a known human B-cell protooncogene that serves a crucial role in lymphocyte development [1]. It is expressed at low levels, mainly in the nuclei of B cells in the germinal centers and mantle zones, a subset of cells in the interfollicular areas of tonsil and lymph nodes, and in the splenic marginal zones [2, 3]. The overexpression of the BCL11A gene has been described in 18 cases of CLL/SLL, invariably associated with the t(2,14) translocation [4–10]. This translocation creates an abnormal fusion between the* BCL11A* (2p16.1) and immunoglobulin heavy chain (*IGH*) gene (14q32), resulting in uncontrolled overexpression of the BCL11A protein and therefore favoring leukemogenesis.

In regard to the* BCL11A* gene location, it was initially mapped to chromosome 2p13, where some cases of lymphoblastic leukemia had shown chromosomal translocations [11]. Subsequently, Menzel et al. stated the location of this gene as 2p15 [12]. It is currently accepted that* BCL11A* gene maps to chromosome 2p16.1 [1].

## 2. Case Presentation

A 49-year-old African American man with history of chronic back pain presented to the ER with three-week history of worsening lower back and bilateral leg pain. He was afebrile and normotensive on presentation with physical examination findings that were notable for tenderness in the lower lumbar spine, nontender enlarged lymph nodes in the cervical, supraclavicular, axillary and inguinal regions, multiple subcutaneous nodules in the skin of the proximal lower extremities; the abdomen was only positive for mild splenomegaly with normal liver size. In the neurologic exam, the motor strength was preserved in all the extremities with normal deep tendon reflexes.

**Figure 1:**
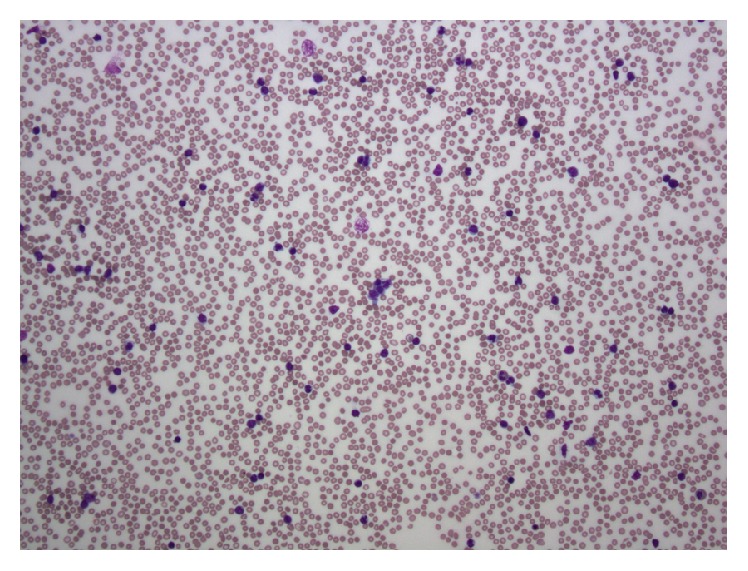
Lymphocytosis in the peripheral blood.

Presenting CBC and chemistries showed white blood cell count (WBC) of 250 K/*μ*L, with 1% neutrophils and 99% lymphocytes, hemoglobin, 6.8 g/dL, and platelet count of 9 K/*μ*L. BMP, LFTs, DIC panel, HIV, and viral hepatitis panel were normal. Tumor lysis panel was only significant for elevated LDH 1008. Peripheral smear showed marked mature lymphocytosis, composed of medium sized cells with mature clumped chromatin, round nuclear contours, moderate cytoplasm, severe thrombocytopenia and hypochromic, and normocytic anemia (Figure 1). Flow cytometry identified 85% of total events as CD19 and CD20 positive B-cells coexpressing CD5, CD23 (heterogeneous), CD200, and kappa light chain. CD38 was expressed in >30% of clonal B-cells. They were negative for CD10, FMC7, and CD34. CT scans revealed cervical lymphadenopathy involving levels I through V bilaterally, as well as extensive lymphadenopathy in the bilateral axilla, inguinal, and external iliac chains. MRI of the spine revealed diffusely abnormal T1 marrow signal, indicative of a marrow replacement by leukemia; no spinal canal stenosis or nerve root impingement was found.

**Figure 2:**
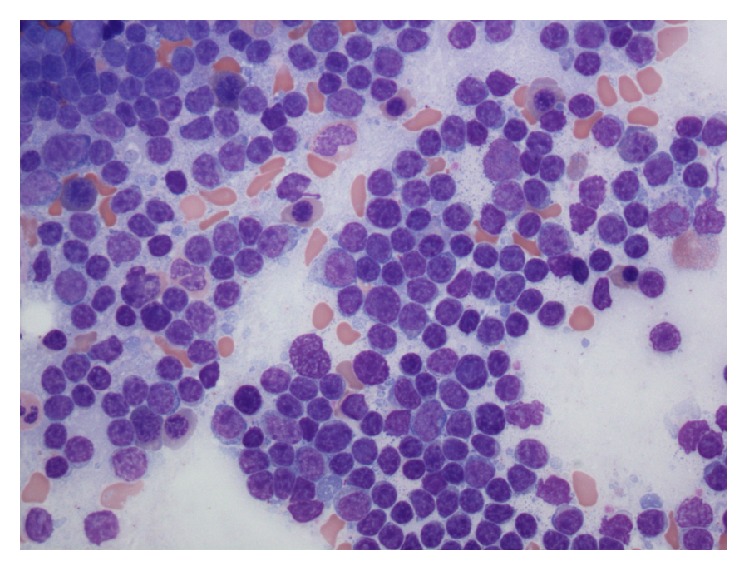
Bone marrow aspirate depicts the marked increase in lymphocytes. They are uniformly small with mature chromatin, scant cytoplasm, and inconspicuous nucleoli.

**Figure 3:**
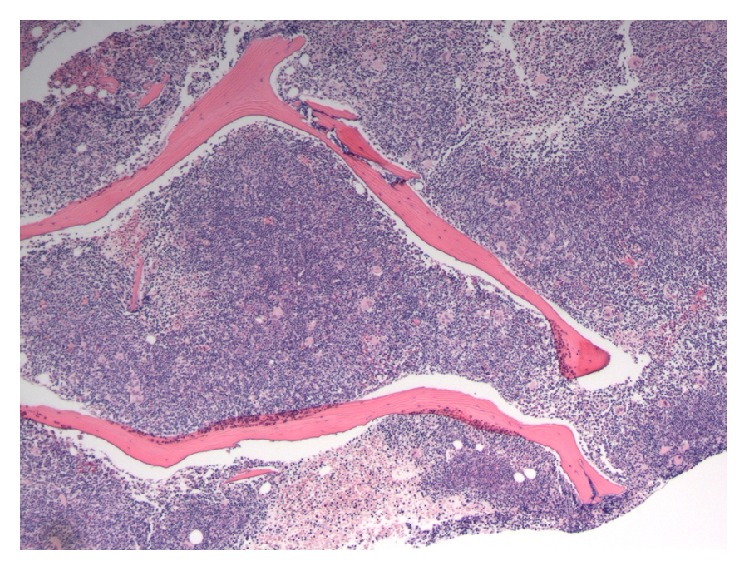
Diffuse involvement of the bone marrow by CLL as shown on this core biopsy specimen.

**Figure 4:**
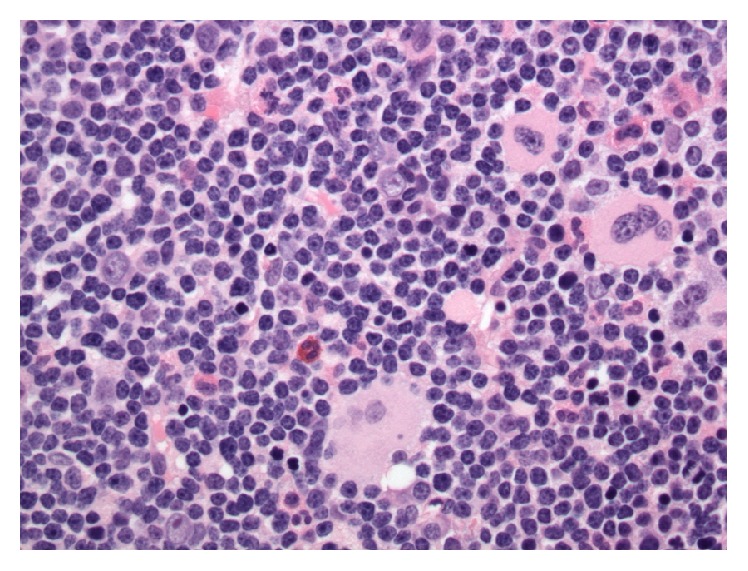
High-power view of the biopsy depicting the markedly increased number of small, mature-appearing lymphoid cells.

**Figure 5:**
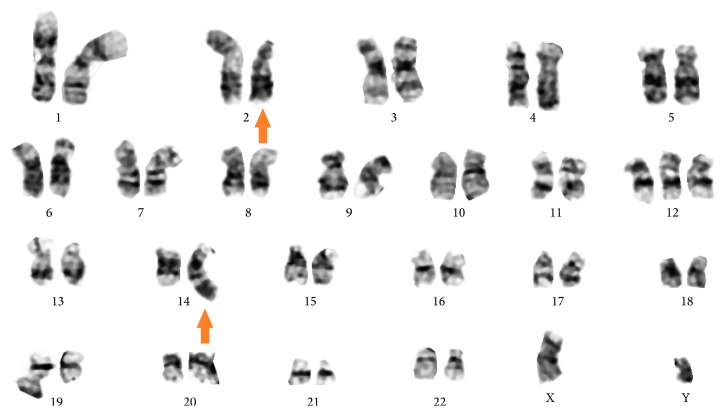
Cytogenetics showing translocation (2;14)(p16;q32). Orange arrow.

Bone marrow biopsy and aspirate showed a markedly hypercellular marrow for age (95% cellularity), essentially replaced by small, mature-appearing lymphoid cells in a diffuse pattern of distribution. The disease burden was estimated at about 90–95% of marrow space, with severely decreased erythropoiesis and granulopoiesis. Megakaryocytes were increased in number and showed adequate morphology (Figures 2, 3, and 4). FISH revealed trisomy 12 and gain of 14q32 (IGH) in 67% and 27% of analyzed nuclei, respectively, as well as gains of 6q, 11q22.3 (ATM), 13q, and 17p13.1 (TP53) in a low number of nuclei (<10%). Conventional cytogenetics demonstrated an abnormal male karyotype: 47,XY,t(2;14)(p16;q32),+12[cp11]/46,XY[1], significant for the presence of the t(2;14) translocation* (BCL11A-IGH)* and trisomy 12 (Figure 5). Unfortunately insufficient material was available to perform specific FISH to further demonstrate the presence of the* BCL11A-IGH* translocation, but analysis of the oncogenes present in chromosome 2p16 renders* BCL11A *as the only potential candidate with demonstrated oncogenic capacity in CLL/SLL [13].* IGHV* gene mutation analysis was reported as unmutated.

The patient was diagnosed with chronic lymphocytic leukemia/small lymphocytic lymphoma (CLL/SLL) and autoimmune thrombocytopenic purpura (ITP). He was started on IV dexamethasone and after two days, he was treated with fludarabine, cyclophosphamide, and rituximab, with rapid improvement of the cell counts; after 2 weeks, the WBC decreased to 12 K/*μ*L; Hb, 9.1 g/dL; and platelet count, 58 K/*μ*L. At the time of the last clinic appointment, the patient had had 4 cycles of FCR and he was asymptomatic and had achieved complete hematologic response, and all the enlarged lymph nodes and skin nodules disappeared.

## 3. Discussion

We performed a review of the available literature and were able to find 19 cases reported; briefly we will summarize them:

Yin et al. described 6 CLL cases with t(2;14)(p16;q32); these patients had an average age of 49.6 years. All of them had marrow involvement, 4 had absolute lymphocytosis, 4 had lymphadenopathy, and 3 of them had hepatosplenomegaly. All showed atypical lymphocyte morphologic features with plasmacytoid differentiation and irregular nuclei; 3 had increased prolymphocytes. Flow cytometry demonstrated 5 patients with typical and one patient with atypical immunophenotype. All expressed ZAP70; 5 assessed patients had unmutated* IGHV* genes. Karyotyping identified t(2;14)(p16;q32) as the sole abnormality in 1, primary abnormality in 2, and part of a complex karyotype in 3 patients. FISH analysis revealed* BCL11A-IGH* rearrangement in all of them. After chemotherapy, 3 patients died of disease and 3 were still alive after a median follow-up of 80 months [6].

Podgornik et al. described a 45-year-old woman with CLL that had atypical phenotype and an aggressive course; initially she had trisomy 12 as only chromosomal abnormality; she was treated with fludarabine, cyclophosphamide, and alemtuzumab with good partial response; after chemo she achieved 5-year disease-free interval; when the disease recurred she underwent an unrelated allogeneic stem cell transplant. One year later, she developed skin lesions that turned out to be Richter's transformation. Her cytogenetics showed trisomy 12 with concomitant balanced translocations t(2;14)(p13;q32), t(14;19)(q32;q13), and t(18;22)(q21;q11). She was successfully treated with 4 doses of ofatumumab achieving a durable remission [5].

Küppers et al. reported 2 adults and 2 pediatric CLL cases with t(2;14) (p13;q32.3); all of these patients had unmutated* IGHV* genes; interestingly one patient was diagnosed with CLL clinically, but the lymph node biopsy was consistent with lymphoplasmacytic lymphoma/immunocytoma expressing monoclonal IgM. This paper focused on the technical aspects of detecting the specific location of the translocation and described that all the* IGH* breaks occurred within the Sy region, whilst the 2p13 breaks clustered centromeric of a CpG island associated with the 5′ end of the* BCL11A *gene [7].

Satterwhite et al. published 4 CLL cases with (2;14)(p13;q32.3), 2 of them were adults and 2 pediatric cases, and interestingly 3 cases exhibited these translocations as the sole or primary cytogenetic abnormality. The first adult patient was a 62-year-old woman, who presented with generalized lymphadenopathy, splenomegaly, and WBC of 396 K/*μ*L. Cytogenetics showed 46,XX,t(2;14)(p13;q32)[14]/46,idem,t(3;6)(p21;q25),del(11)(q22q23)[2]/46,idem,add(8)(p23)[2] indicating that t(2;14)(p13;q32) was the primary cytogenetic abnormality [8]. She was treated with chlorambucil and subsequently with fludarabine but failed to respond to either and died of progressive disease after 2 years of diagnosis.

The second adult patient presented with generalized lymphadenopathy, hepatosplenomegaly, and a WBC of 38.3 K/*μ*L. The clinical diagnosis of CLL was established. However, a lymph node biopsy was consistent with a lymphoplasmacytic immunocytoma according to the Kiel classification with increased proliferation activity and monoclonal IgM kappa expression. Cytogenetic analysis of both the lymph node and the peripheral blood showed the karyotype: 46,XY,t(2;14)(p13;q32),t(18;21)(p11;q21). After 6 years, the patient presented with clinical progression including lymphadenopathy, B-symptoms, and a WBC of 76.5 K/*μ*L. Histopathology of a repeat lymph node biopsy again revealed lymphoplasmacytic immunocytoma. Chromosomal analysis showed the karyotype: 46,XY,t(2;14)(p13;q32),t(18;21)(p11;q21)[6]/46,idem,t(13;15)(q12;13;q21). This patient died after 13 years of the diagnosis due to progressive disease [9]. He did not describe the clinical findings of the pediatric cases.

Richardson et al. reported two cases of CLL in children with t(2;14)(p13;q32) in 1992. He hypothesized that there may be a potential oncogene located near the 2p13 breakpoint which may have been activated by the t(2;14) translocation in these two cases of CLL [9]. Fell et al. described the first two cases of CLL in children t(2;14) (p13;q32) opening the hypothesis that these cases represent a rare but distinct subgroup of CLL/SLL with a specific cytogenetic change [10].

## 4. Conclusion

Here we report the case of a young male with very high WBC that was found to have CLL/SLL with associated t(2;14) translocation* (BCL11A-IGH)* and trisomy 12. To the best of our knowledge, there have been only 19 cases reported in the literature; all of them had younger age compared to regular CLL patients and 8 patients were pediatric cases; most of them had atypical cytology features and unmutated immunoglobulin heavy chain mutation status and were ZAP70 positive. In addition, the majority of these patients also presented with diffuse lymphadenopathy and elevated WBC and had the t(2;14) translocation as the sole or primary cytogenetic abnormality. Of note, the most frequently associated chromosomal abnormalities in CLL/SLL, such as del 11q, trisomy 12, del 13q, and del 17q, were absent in these patients. Interestingly there were 2 patients that were found to have lymphoplasmacytic lymphoma/immunocytoma with this translocation. We do not have enough available long term survival data to evaluate if the presence of this gene fusion (i.e.,* BCL11A-IHG*) has a poorer or better overall survival, but most of the reported patients have survived for several years with the standard chemotherapy, suggesting that its impact in overall survival may not be important enough to grant more aggressive therapies and/or different follow-up schemes.
